# The Horizontal Raphe of the Human Retina and its Watershed Zones

**DOI:** 10.3390/vision3040060

**Published:** 2019-11-08

**Authors:** Christian Albrecht May, Paul Rutkowski

**Affiliations:** Department of Anatomy, Medical Faculty Carl Gustav Carus, TU Dresden, 74, 01307 Dresden, Germany; pcr.11md@gmail.com

**Keywords:** anatomy, choroid, development, human, retina, vasculature

## Abstract

The horizontal raphe (HR) as a demarcation line dividing the retina and choroid into separate vascular hemispheres is well established, but its development has never been discussed in the context of new findings of the last decades. Although factors for axon guidance are established (e.g., slit-robo pathway, ephrin-protein-receptor pathway) they do not explain HR formation. Early morphological organization, too, fails to establish a HR. The development of the HR is most likely induced by the long posterior ciliary arteries which form a horizontal line prior to retinal organization. The maintenance might then be supported by several biochemical factors. The circulation separate superior and inferior vascular hemispheres communicates across the HR only through their anastomosing capillary beds resulting in watershed zones on either side of the HR. Visual field changes along the HR could clearly be demonstrated in vascular occlusive diseases affecting the optic nerve head, the retina or the choroid. The watershed zone of the HR is ideally protective for central visual acuity in vascular occlusive diseases but can lead to distinct pathological features.

## 1. Introduction

The horizontal raphe (HR) was first described in the early 1800s as a horizontal demarcation line that extends from the macula to the temporal Ora dividing the temporal retinal nerve fiber layer into a superior and inferior half [1]. In 1903 Leber’s illustration of the uveal vascular supply showed a similar horizontal separation of the superior and inferior choroidal vessels along the horizontal meridian [2]. Polyak implied that the nasal portion of the HR from the macula to the optic nerve head could be imagined, and this portion was demonstrated seven years later by Posner and Schlossman using perimetric studies in glaucoma patients [3,4]. There was, however, no agreement that the HR separates the superior and inferior retinal circulation [5]. Vrabec histologically examined the HR in the temporal macular region and found nerve fibers bundles crossing the HR for short distances [6]. He agreed with previous investigators that a true anatomic HR did not exist, but referred to it enigmatically as a “physiologic” HR.

Renewed interest in the HR resulted from innovative retinal imaging instruments with advanced software analysis in in vivo retinae. The confocal scanning laser ophthalmoscope and the optical coherence tomography (OCT) instruments using interface reflectance analysis can measure the thickness of the retinal nerve fiber layer surrounding the optic nerve head, and the latter can also measures the nerve fiber layer and retinal ganglion cell layer thickness surrounding the macula. Advanced optics confocal scanning laser ophthalmoscopy (AOSLO) can measure the various retinal layers temporal to the macula. These instruments objectively measure the nerve fiber layer thickness and defects in the nerve fiber layer thickness that exists adjacent to the optic nerve head and at the macula in aging and glaucoma [7,8,9,10,11]. However, at this time, the measurements of the peripapillary nerve fiber layer loss in glaucoma are more accurate than those measurements of the nerve fiber layer loss performed at the macula [7,12]. The peripapillary nerve fiber layer measurement theoretically measures 100% of the retinal ganglion axons, but the macular OCT measures only 34% of the total retinal ganglion axons [12,13]. It was reported erroneously that the macula contains as high as 50% of the total retinal ganglion axons [12]. One study using AOSLO found the width of the HR measured 1 mm temporal to the fovea to be approximately 110 µm, and the nerve fiber layer was found to be less than 30 µm in thickness [14]. The adult nerve fiber layer thickness is 50 µm from the nasal to the fovea [15]. Several studies demonstrated that the temporal HR does not follow exactly the horizontal meridian, but is inclined, on average, 10 degrees above the horizontal meridian [8,14,16]. Both the confocal scanning laser ophthalmoscope and the OCT play a clinical role in the diagnosis of diseases affecting the nerve fiber layer and the HR. At present, the AOSLO remains primarily a research instrument [15].

Although the concept of a temporal HR is accepted, its length, width, and depth have not been adequately researched. We performed a literature review using the key words: Temporal raphe, horizontal raphe, nerve fiber layer development, and retinal ganglion cell axon by pooling single published data over the last four decades.

Understanding the complex developmental anatomy of the retina and choroid in the fetus and childhood allows one to better understand the HR’s origin, composition, and extent along the horizontal meridian, along with the role it plays in the clinical picture of adult occlusive vascular disease involving the optic nerve head, the retina, or the choroid.

## 2. Developmental Aspects of the Human Horizontal Raphe

### 2.1. Molecular Signals Involved in Fetal Nerve Fiber Layer Axon Guidance

Although little data exists on nerve fiber layer axon guidance in human fetal tissue, fetal molecular guidance signals are considered similar in most mammals. Numerous studies on mice revealed some key players involved in the different steps of axon growth into the nerve fiber layer, their elongation toward the optic nerve head, and their passage along the retrobulbar optic nerve [17,18]. The slit-robo cell signaling pathway prevents the axons from growing into the outer retina [19,20,21]. Secreted frizzled related proteins support the slit-robo inhibition of axon sprouting into the outer retina and modulates the fasciculation of the axons during their elongation toward the optic nerve head [22,23]. The ventral-dorsal orientation of the retina is maintained by a number of signals, including the chondroitin sulphate proteoglycan [17], the different quadrants of the retina by the ephrin-protein-receptor system [18], the higher inferior to lower superior gradient by the EphB receptor proteins, the opposite high-low gradient pattern by the ephrin-B proteins, and a similar opposing gradient along the nasal-temporal axis by EphA receptor proteins and ephrin-A molecules [24]. The direction of the NFL axons toward the optic nerve is triggered, among others, by netrin-1 and heparan sulphate proteoglycans. Gradients of the nasal-temporal axis orientation occurring at both the posterior pole and peripheral retina were confirmed by transcriptome analysis [25]. However, no markers were mentioned to establish a superior-inferior gradient. Transcriptome analysis demonstrated a timeline development of each fetal retinal layer occurring between the 19th and 21st week of gestation (wog) with no mention of the HR [26]. It is therefore tempting to speculate that the development of the horizontal raphe is not induced by specific molecules, but rather by other morphological factors discussed below.

### 2.2. Morphological Organization of the Human Nerve Fiber Layer

During the sixth to eighth wog, the retinal ganglion cells differentiate first in the future macular region of the optic cup [27]. These retinal ganglion cells sprout axons that grow toward the optic stalk and, eventually, to their target cells in the brain. These retinal ganglion cell axons will become the future papillomacular bundle. The number of retinal ganglion cell axons increases rapidly during the first phase of fetal eye growth, reaching a maximum number of approximately 3.7 million axons during the 16th to 17th wog [28,29]. Following this first period of fetal development, the eye growth stagnates, and the number of retinal ganglion cell axons decline precipitously to 1.1 million (roughly the adult number) around the 29th wog. During this second phase of fetal development, there is an approximate 70% loss (apoptosis) of the retinal ganglion cell axons, primarily in the retinal periphery. Comparative studies in various mammals revealed that initially the retinal ganglion cells and their axons are evenly distributed throughout the retina. A similar high retinal ganglion cell axons apoptosis occurs, but this apoptosis is found in all retinal regions, unlike in humans, where this apoptosis primarily occurs in the retinal periphery [30,31,32]. This apoptosis is thought to be initiated by a lack of synaptic integration of retinal ganglion cells that project their axons toward the higher brain centers [33,34]. Some subpopulations of retinal ganglion cells and their axons seem to be excluded from this loss [35]. The distinct topography of adult retinal ganglion cell density is also related to nonuniform expansion of the retina due to mechanical forces during late fetal growth and the marked eye growth after birth [36,37]. The course of the developing nerve fiber layer arcuate axon bundles also affects the adult position of the four major branches of the central retinal artery. At this point in fetal development, there are no morphological features pointing to the existence of the horizontal raphe [38,39,40].

### 2.3. The Impact of the Choroidal Development on Defining the Horizontal Raphe

Since neither molecular signals nor definite retinal structures can sufficiently explain the creation of the HR, some local intrinsic factors must be considered. Perhaps the most striking structural influence can be found in the developing choroidal vasculature [41,42]. The earliest choroidal vascular condensations occur around the optic cup during the fouth wog [27]. During the seventh wog, the long posterior ciliary arteries arise from the ophthalmic artery and grow forward in the horizontal meridian toward the immature ciliary body. Subsequently, in the eighth wog, the short posterior ciliary arteries begin to invade the immature choriocapillaris [5]. By the fourth month of gestation, their branches connect with the lobules of the maturing choriocapillaris [42,43]. Both of the long posterior ciliary arteries divide the developing immature choroid along the horizontal meridian, preventing the superior precapillary branches of the short posterior ciliary arteries from connecting with inferior branches across the horizontal meridian. Only at the level of the choroidal capillaries is there a segmental connection across the horizontal meridian [44]. This is in contrast to the rodent’s eye, where the superior and inferior hemispheres of the choriocapillaris are clearly separated by the nasal and temporal long posterior ciliary arteries [45].

Summarizing the data concerning the origin of the HR, we speculated that the initial cause for HR formation comes from the choroid and is morphologically stabilized due to biochemical factors guiding the developing nerve fibers and blood vessels.

## 3. Composition and Extend of the Horizontal Raphe

### 3.1. Postnatal Nerve Fiber Layer Development

The first three years of life result in rapid eye growth that involves the peripheral retina more than the retina at the posterior pole. This phase of nonuniform expansion of the retina, together with the greater fetal retinal ganglion cell apoptosis in the peripheral retina, leads to a decreased density of retinal ganglion cells and their axon bundles in the peripheral nerve fiber layer. At birth, the papillomacular bundle is of even thickness in an arc along the temporal side of the optic nerve head [46]. As the papillomacular bundle approaches the optic nerve head, its axon bundles thicken. To evaluate this neonatal thickness of the nerve fiber layer, a handheld spectral domain optical coherence tomograph was used. One study of 50 full-term neonates using this device found the papillomacular bundle thickness to be 88 µm at 1.1mm and 64 µm at 1.7 mm from the center of the optic nerve head [46]. Another study of children ages 0–13 years found a decrease after birth in papillomacular bundle thickness measured 1.7 mm from center of the optic nerve head. At birth, it measured 70 µm, but decreased to 46 µm at 12 months of age. Thereafter, the nerve fiber layer thickened to 55 µm at 13 years of age [47]. During this time period, from birth to 13 years, the distance from the optic nerve head to the fovea increased only 0.5 mm (12%), whereas the peripheral retina increased by 48% [48,49]. Two additional studies in older children ages six to 13 and six to 16 years measured temporal peripapillary retinal thickness at 76.5 µm and 69.3 µm [50,51]. Both of these studies show little change in papillomacular bundle thickness between ages 6 to 16 years. Variability in nerve fiber layer thickness was found in refractive errors, age, axial length [52,53], ethnic origin [46,54,55], and ocular magnification [52,56]. The nerve fiber layer is consistently thin in the fovea region and changes little with aging [57]. The average papillomacular bundle thickness in adults measured by OCT is approximately 70 µm, but by histological examination, it measures 49 µm [58,59,60]. During this postnatal eye development, the HR lengthens along with the growth of the eye. Its pathway passes from the optic nerve head through the fovea centralis to the temporal ora serrata and nasally from the optic nerve head to the nasal ora serrata. The temporal HR forms an angle with a line connecting the optic nerve head with the center of the fovea that can vary, the average angle being 10 degrees superior to the horizontal meridian [61,62].

### 3.2. Mature Human Retinal Nerve Fiber Layer

The nerve fiber layer is composed of multiple bundles of retinal ganglion cell axons and increases in thickness as these bundles approach the optic nerve head. The retinal ganglion cell axon bundles surrounding the fovea measure 60–70 μm in diameter and are similar in size to the diameters of the capillary nets perfusing them [6]. The retinal ganglion cell axon bundles provide a rough topographic representation within the intraocular part of the optic nerve according to the quadrant they originate in [63]. In monkeys, Minckler showed that the most peripheral retinal ganglion cells give rise to axons bundles that lie deepest in the nerve fiber layer as these axon bundles approach the optic nerve head [64]. At the optic nerve head, these peripheral axon bundles enter the peripheral temporal optic nerve head, as first mentioned by Leber [65]. The retinal ganglion cells located in the peripapillary zone adjacent to the optic nerve head send their axon bundles upward and pierce the nerve fiber layer, occupying a position under the internal limiting membrane. From this position, these axon bundles enter the center of the optic nerve head. Other investigators found similar results in primates [66,67,68]. Only one investigation was performed on the retinotopic organization of human nerve fiber layers [69,70]. They used crystals of carbocyanine dye injected into the peripheral retina as a means of tracing axons within the nerve fiber layer. They demonstrated that the majority of retinal ganglion cell axons bundles arising in the periphery and peripapillary regions are located in the center of the proximal arcuate nerve fiber layer bundles and enter the superior and inferior pole of the optic nerve head in the form of a wedge, similar to a slice of pie. Along their pathway, the individual axons show varicosities rich in mitochondria and have interconnections with desmosomes, with each other, and with surrounding glia cells [71].

### 3.3. The Adult Nerve Fiber Layer Perfusion

The perfusion of the retinal nerve fiber layer along the HR is complex, as it is derived from different capillary beds in different regions of the retina [72]. Generally, the nerve fiber layer perfusion is similar to that of the retinal ganglion cells, except around the optic nerve head (peripapillary) and the fovea (perifoveal). Beyond these two regions, the nerve fiber layer, along with the inner retina, is perfused by the retinal superficial capillary bed. It receives assistance from the deep capillary bed when the diameters of the superficial capillary nets are greater than 90 μm. Beyond the termination of the deep capillary bed at 32–35° temporally and 42–45° nasally from the fovea, the nerve fiber layer and the inner retina are perfused solely by the superficial capillary bed when the diameters of their capillary net do not exceed 90 µm. When their capillary net diameters exceed 90 µm, their centers and the overlying nerve fiber layer are perfused by the choriocapillaris. The choriocapillaris perfuses full retinal thickness at the capillary-free zones of the fovea and the retina for a short distance posterior to the ora serrata. The peripapillary nerve fiber layer bundles entering the superior and inferior poles of the optic nerve head and those nerve fiber layer entering temporally from the macula receive their primary perfusion from the radial peripapillary capillaries with a minor assistance from the superficial capillary bed. This radial peripapillary capillary bed perfuses up to 8 mm of the nerve fiber layer temporal to the optic nerve head but avoids the fovea [73]. The narrow demarcation line of the HR broadens at the retinal foveal avascular zone from a width of 110 µm to an oval enlargement measuring approximately 500 µm in diameter. The submacular and peripapillary choriocapillaris exhibit a poorly defined lobular pattern (dense honeycomb-like structure,) whereas the remainder of the peripheral choriocapillaris has a lobular pattern: 86% of the lobules are perfused by a peripheral lobular arteriole and 14% by a central lobular arteriole [74,75].

### 3.4. The HR Watershed Zones (WSZs)

A watershed zone is the border between the territories of distribution of any two end arteries [76]. The HR WSZ exists on either side of the HR extending from the temporal Ora serrata through the fovea to the optic nerve head. Nasally, it passes from the optic nerve head to the nasal Ora serrata. The choroidal HR WSZ is located immediately exterior to the retinal HR WSZ [2]. In healthy patients, the retinal and the choroidal vascular hemispheres perfuse the various levels of the HR in unison. However, in vascular occlusive diseases affecting either the retina or the choroid, one HR WSZ may be compromised, leaving the other WSZ with normal perfusion. The occlusive vascular diseases affecting the retina are obvious and can be demonstrated in their acute phase with fundus photography, fundus angiography, Humphrey visual field analysis, and even with the naked eye and an ophthalmoscope. However, vascular occlusive diseases involving the choroid are hidden from view by a normal functioning retina and can only be diagnosed by an abnormal visual field accompanied with a normal peripapillary retinal nerve fiber layer and macular retinal ganglion cell layer. One exception is the occlusion of a cilioretinal artery that produces an infarcted area of the retina.

## 4. Clinical Cases Demonstrating the Presence of the HR

### 4.1. Arteritic Ischemic Optic Neuropathy

**Figure 1:**
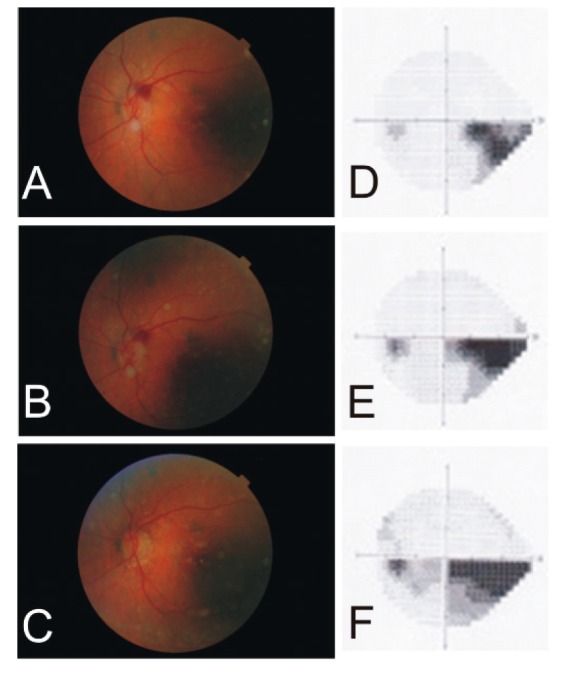
A series of fundus photographs and Humphrey visual fields (24–2) demonstrating the progress of an arteritic ischemic optic neuropathy in the left eye of a 91-year-old female. Note the big red spot next to the optic nerve (**A**), which became smaller after one week (**B**) and disappeared after one month (**C**). The initial scotoma of the inferior temporal quadrant (**D**) increased continuously after one year (**E**) and three years (**F**).

A 91-year-old female demonstrated progression of her visual field defect from the temporal to the nasal HR at the posterior pole (Figure 1). The optic nerve head ischemia involved the superior arcuate nerve fiber bundle and gradually involved the superior half of the papillomacular bundle. Her visual acuity remained 20/30, and her erythrocyte sedimentation rate remained within a normal range on prednisone as her HR scotoma gradually enlarged along the inferior HR. 

### 4.2. Cilio-Retinal Artery Occlusion

**Figure 2:**
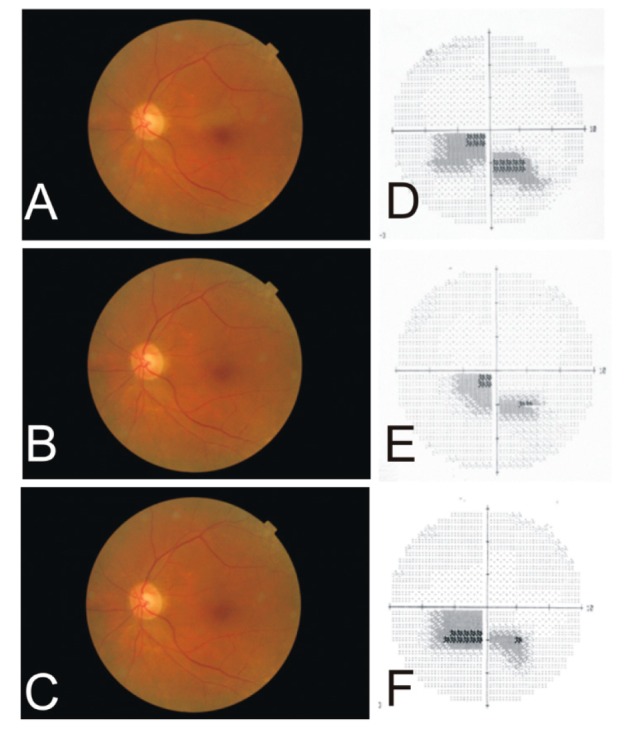
A series of fundus photographs and corresponding Humphrey visual fields (10–2) demonstrating the development of a cilio-retinal artery occlusion in the left eye of an 81-year-old female. The initial scotoma (**A**,**D**) in the inferior visual field improved somewhat after three months (**B**,**E**) but remained constant after four years (**C**,**F**).

An 81-year-old female developed a sudden onset of a painless scotoma in front of her left eye. The retinal edema associated with this scotoma resulted from occlusion of a cilioretinal artery arising on her temporal optic nerve head (Figure 2). Her Humphrey visual field (10–2) showed the scotoma paralleling the HR. This occlusion was demonstrated by the fluorescein angiograph as “box cars” during retinal consultation. Although her vision remained corrected to 20/25, there was late blunting of the superior foveal rim due to the loss of retinal ganglion cells on retinal photographs.

### 4.3. Branch Retinal Artery Occlusion

**Figure 3:**
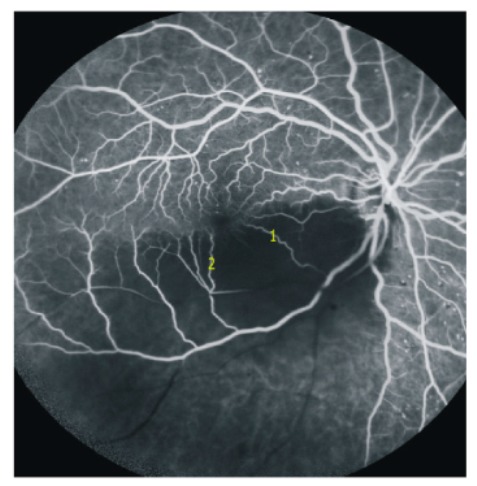
Retinal angiogram image (Figure 3) as a courtesy of Dr. Jeremy Chess, New York. Note the occluded retinal artery branch (black vessel in the inferior temporal quadrant). The functioning retinal capillary anastomoses fill the precapillary venules of the ischemic side (1 and 2 as examples). The capillary ring around the avascular foveal region appears complete.

Unfortunately, we have only this retinal angiogram image (Figure 3) as a courtesy of Dr. Jeremy Chess, New York, and no clinical data. This branch retinal artery occlusion demonstrates the functioning retinal capillaries anastomoses at the HR and perfusing precapillary venules of the ischemic half of the ipsilateral retinal vascular hemisphere. The retinal capillary bed, adjacent to the foveal avascular zone, was filled on both the non-ischemic and the ischemic side through the dense retinal capillary “anastomotic ring” surrounding the foveal avascular zone. There was some retrograde filling of the retinal arterioles from the nonischemic precapillary venule across the capillary bed of the HR. 

### 4.4. Choroidal Retinal Artery Occlusion

**Figure 4:**
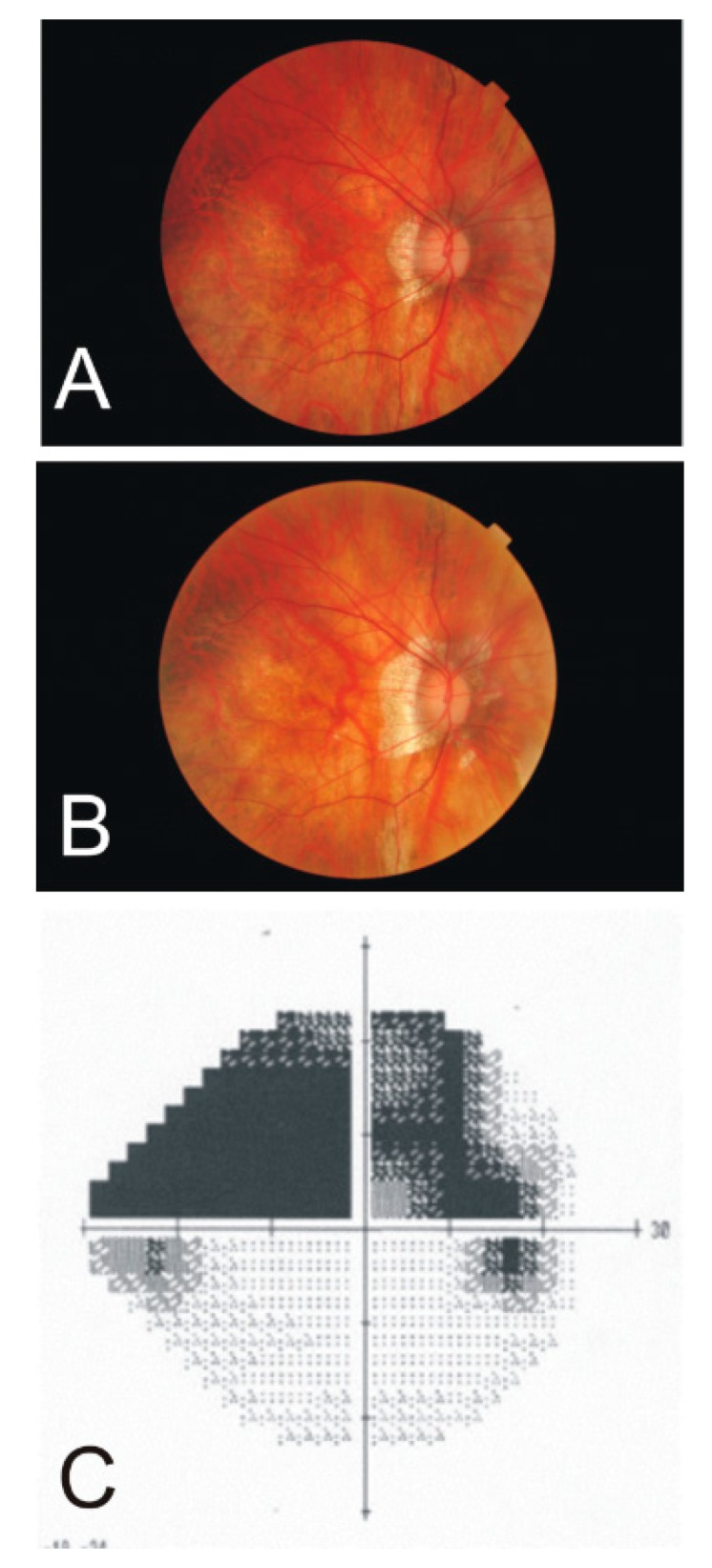
Fundus photographs of the right eye of a female with high myopia at age 45 (**A**) and age 57 (**B**). Note the increasing peripapillary defect suspecting a choroidal retinal artery occlusion. The Humphrey visual field (24–2) measurement at age 57 (**C**) shows a superior hemiretinal visual field defect (dark grey areas).

On retinal exam, a 57-year-old female bilateral pseudophake was found to have an abnormally appearing optic nerve head in the right eye. A large superior arcuate visual field defect was found abutting the HR (Figure 4). Retinal photographs of the optic nerve head of the right eye were also existent from 12 years earlier. Clinically, her visual acuity remained 20/20 to 20/25 in the right eye. The axial length at the time of her cataract surgery 13 years ago (06/21/2005) was 31.9 mm for the right eye and 31.3 mm for the left eye. Repeated actual axial length was 33.66 mm for the right eye and 33.01 mm for the left eye. This is an increase in retinal area of 10.4% in the right eye and 10.6% in the left eye.

### 4.5. Hemiretinal Vein Occlusion

**Figure 5:**
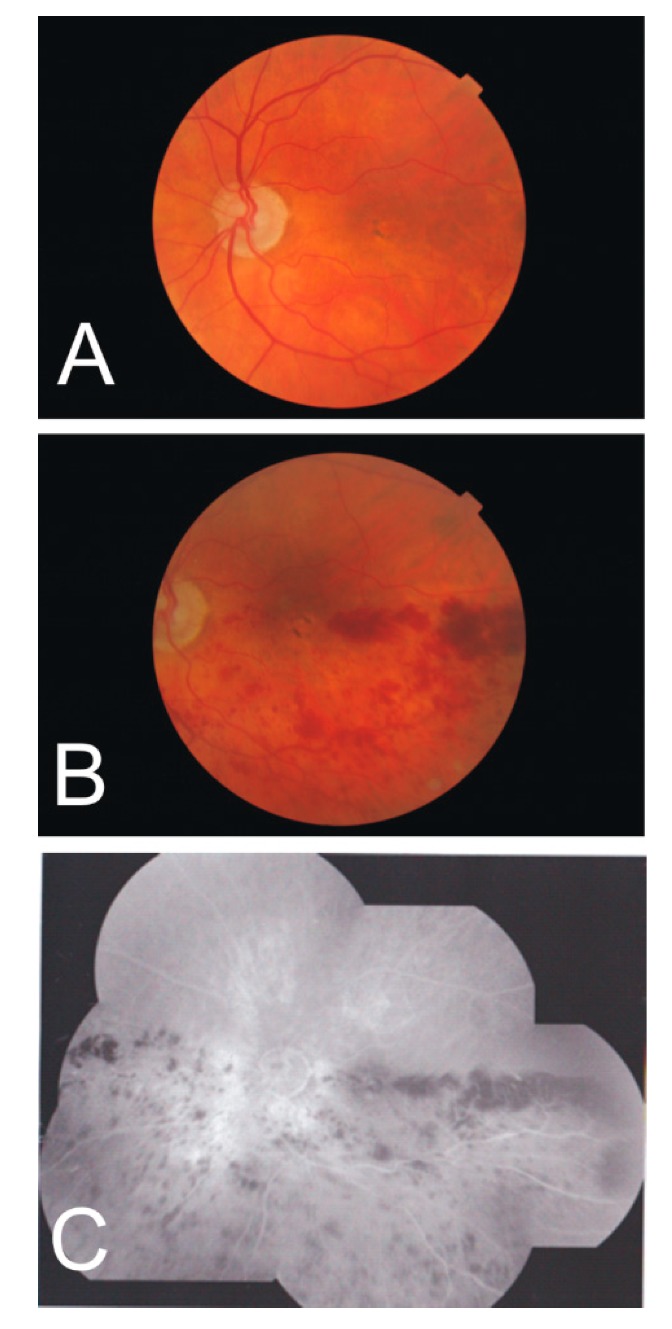
Fundus photographs of a 92-year-old male left eye at the onset of vein occlusion (**A**) and 10 months later (**B**), revealing the development of hemorrhage accumulations somewhat recessing the fovea centralis. (**C**) A photo collage at the second time point shows the symptoms in both the temporal and nasal quadrants of the inferior half.

A 93-year-old male with controlled capsular glaucoma and moderate age-related retinal macular degeneration (dry) in both eyes developed a vein occlusion in his left eye that progressed to inferior hemiretinal vein occlusion (Figure 5). Retinal hemorrhages accumulate along the inferior horizontal meridian, delineating the HR in both the temporal and nasal quadrants. This accumulation of hemorrhages inferior to the fovea is minimized due to the dense retinal capillary “anastomotic ring” surrounding the foveal avascular zone.

## 5. Discussion and Conclusions

**Figure 6:**
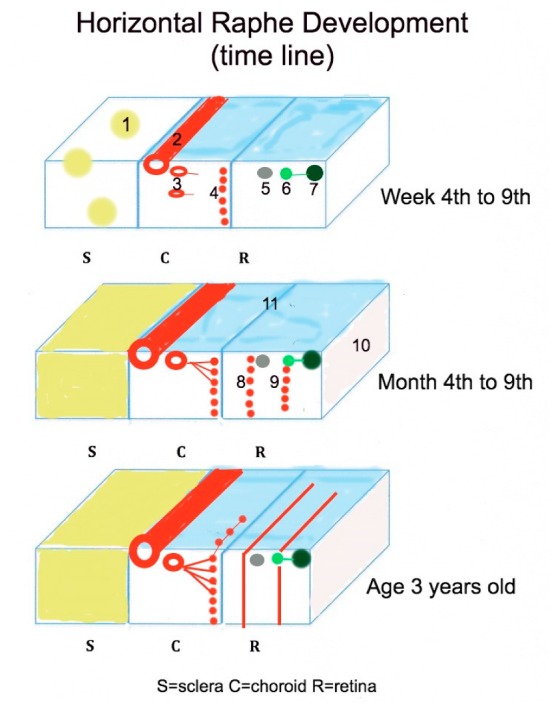
Schematic summary of the structures influencing the development of the horizontal raphe. **1** = scleral condensation, **2** = long posterior ciliary artery appearance, **3** = short posterior ciliary artery, **4** = immature choriocapillaris, **5** = Müller cells, **6** = retinal ganglion cells, **7** = retinal nerve fiber layer, **8** = immature deep retinal capillary bed, **9** = immature superficial retinal capillary bed, **10** = inner limiting membrane, **11** = chorio-retinal interface.

The formation of the HR in humans occurs with the growth of the long posterior ciliary arteries along the horizontal meridian during the seventh week of gestation and is summarized in Figure 6. This growth of the arteries divides the developing fetal papillomacular bundle into both superior and inferior axon groups. However, some errant axon nerve fibers cross the horizontal meridian to short distances in the macular region, resulting from the one-week delay in growth onset of the long posterior ciliary arteries compared to the retinal vessels. These errant axon bundles appear to disappear in the adult [11]. By the fourth month of gestation, the choriocapillaris begins to assume a more adult form as their lumens increases in diameter to 40–60 µm [77]. The preganglionic branches of the short posterior ciliary arteries connect to the capillary lobules at the posterior pole and choroidal periphery [74,75]. At this stage in fetal development, these lobules communicate across the horizontal raphe. providing the main perfusion of the developing retina.

The fetal retinal capillary bed development begins with first the superficial capillary bed in the fourth month of gestation, followed, in order, by the radial peripapillary capillary bed, the deep capillary bed, and the creation of the foveal avascular zone in the ninth month of gestation [78,79]. All three of these retinal capillary beds remain an anastomosing honeycomb of immature vessels that fail to encompass the far temporal periphery at birth [80]. After birth, the retinal vessels mature, with only the retinal capillaries crossing the retinal HR. The retinal precapillary arterioles and venules do not cross the raphe due to probable adequate retinal tissue oxygen levels at the raphe. 

As retinal vascular maturation continues on both sides of the HR, one finds precapillary arterioles perfusing some retinal capillaries that cross the HR and become contralateral precapillary venules. These venules drain into contralateral retinal veins and finally into the contralateral central retinal vein. This filling of central retinal veins by some venous blood from the contralateral retinal vascular hemisphere can be demonstrated in the ischemic side of the branch retinal artery occlusion, and particularly, the marked accumulation of hemorrhages along the inferior nasal and temporal HR in the hemiretinal vein occlusion. At the fovea, there is a paucity of HR hemorrhages due to the adequate venous drainage by the dense retinal capillary bed anastomoses surrounding the avascular zone. 

Around the age of three, the raphe is composed of different components: A barrier of vertical silo-like structures measuring 50–70 µm in diameter, passing from the external limiting membrane to the internal limiting membrane, containing nerve fiber bundles and neurons from the retinal photoreceptors to the retinal ganglion cells, shrink-wrapped by Mueller’s glial processes [6,16,81]; a nerve fiber layer consisting of adjacent mainly parallel running bundles of the superior and inferior retinal hemispheres; anastomosing capillary beds of separately the choroid and retina at various levels of the HR, bordered by the precapillary arterioles of the retinal and choroidal circulation. The Raphe extends from the internal limiting membrane to the underlying sclera. It is a pseudo-structural interface located along the horizontal meridian that divides the posterior segment of the eye into a superior and inferior vascular hemisphere. 

In early fetal development, molecular signals define a retinal nasal-temporal axis orientation, but no markers have been found that established a retinal superior–inferior axis orientation. This deficiency may play a role in the direction of growth of the long posterior ciliary arteries in the vertical plane and explain the varying angle that the adult HR forms with the anatomical horizontal meridian.

Images of retinal and cilioretinal arteries occlusions from emboli are well-documented in the literature. Similar embolic disease or arteriosclerosis affecting the vessels of the optic nerve head or the choriod occur, but these occlusions remain difficult to demonstrate in vivo. In Case 1 and Case 4, we suspected that the visual field defect was caused by occlusion of a posterior ciliary artery. In Case 1, the swollen superior temporal quadrant of the optic nerve head demonstrated the area of ischemic infarct but not the posterior ciliary artery occlusion. The best explanation is that this ischemia was caused by occlusion of one posterior ciliary artery in an eye with four posterior ciliary arteries, according to Hayreh [76].

In Case 4 of suspected posterior ciliary artery occlusion, the triad of intact RNFL analysis, a mainly intact RGC layer, and a marked superior arcuate field defect could only occur with a normal functioning superficial retinal capillary bed. The arcuate visual field defect was the result of apoptosis of photoreceptors not the retinal ganglion cells. This choroidal was probably due to occlusion of one posterior ciliary artery in an eye with three posterior ciliary arteries, where the vertical watershed zone occurred temporal to the optic nerve head [76]. What role, if any, did the stretching of the eyeball play in the posterior ciliary artery occlusion and the slightly abnormal appearance of the optic nerve head? 

The preservation of central visual acuity is often achieved in occlusive vascular disease of retina and choroid due to the dense retinal anastomosing capillary ring surrounding the foveal avascular zone and due to the drainage of the choroidal submacular capillary bed [74,75]. This protection of central visual acuity is particularly important today as the lifespan of humans increases with its attendant increase of vascular occlusive disease associated with obesity, diabetes, and hypertension.
